# (*E*)-Ethyl *N*′-[1-(2-hydroxy­phen­yl)ethyl­idene]hydrazinecarboxyl­ate

**DOI:** 10.1107/S1600536808025191

**Published:** 2008-08-16

**Authors:** Bo Gao

**Affiliations:** a Marine College, Zhejiang Institute of Communications, Hangzhou 311112, People’s Republic of China

## Abstract

In the mol­ecule of the title compound, C_11_H_14_N_2_O_3_, the dihedral angle between the benzene ring and the hydrazinecarboxyl­ate mean plane is 3.65 (12)°. Intra­molecular C—H⋯N and O—H⋯N hydrogen bonds result in the formation of a nearly planar six-membered ring, which is oriented at a dihedral angle of 2.38 (3)° with respect to the benzene ring, and a five-membered ring having an envelope conformation. In the crystal structure, inter­molecular N—H⋯O and C—H⋯N hydrogen bonds link the mol­ecules. There is a C—H⋯π contact between the benzene ring and the methyl group of the ethyl substituent.

## Related literature

For general background, see: Parashar *et al.* (1988 ▶); Hadjoudis *et al.* (1987 ▶); Borg *et al.*, (1999 ▶). For a related structure, see: Gao (2008 ▶).
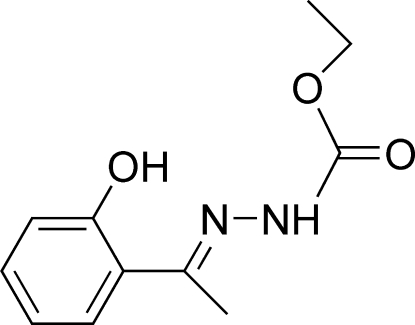


## Experimental

### 

#### Crystal data

C_11_H_14_N_2_O_3_
*M* *_r_* = 222.24
Monoclinic, 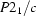

*a* = 8.0841 (7) Å
*b* = 23.052 (2) Å
*c* = 6.6019 (6) Å
β = 111.584 (3)°
*V* = 1144.03 (18) Å^3^
*Z* = 4
Mo *K*α radiation
μ = 0.10 mm^−1^
*T* = 273 (2) K
0.28 × 0.24 × 0.23 mm

#### Data collection

Bruker SMART CCD area-detector diffractometer
Absorption correction: multi-scan (*SADABS*; Bruker, 2002 ▶) *T*_min_ = 0.973, *T*_max_ = 0.981
12167 measured reflections
2018 independent reflections
1360 reflections with *I* > 2σ(*I*)
*R*_int_ = 0.089

#### Refinement

*R*[*F* ^2^ > 2σ(*F* ^2^)] = 0.040
*wR*(*F* ^2^) = 0.117
*S* = 1.05
2018 reflections
149 parameters
H-atom parameters constrained
Δρ_max_ = 0.21 e Å^−3^
Δρ_min_ = −0.12 e Å^−3^

### 

Data collection: *SMART* (Bruker, 2002 ▶); cell refinement: *SAINT* (Bruker, 2002 ▶); data reduction: *SAINT*; program(s) used to solve structure: *SHELXS97* (Sheldrick, 2008 ▶); program(s) used to refine structure: *SHELXL97* (Sheldrick, 2008 ▶); molecular graphics: *SHELXTL* (Sheldrick, 2008 ▶); software used to prepare material for publication: *SHELXTL*.

## Supplementary Material

Crystal structure: contains datablocks I, global. DOI: 10.1107/S1600536808025191/hk2507sup1.cif

Structure factors: contains datablocks I. DOI: 10.1107/S1600536808025191/hk2507Isup2.hkl

Additional supplementary materials:  crystallographic information; 3D view; checkCIF report

## Figures and Tables

**Table 1:** Hydrogen-bond geometry (Å, °) *Cg*1 is the centroid of the benzene ring.

*D*—H⋯*A*	*D*—H	H⋯*A*	*D*⋯*A*	*D*—H⋯*A*
O1—H1⋯N1	0.82	1.85	2.5604 (18)	145
N2—H2*A*⋯O2^i^	0.86	2.07	2.9175 (18)	167
C8—H8*A*⋯N2	0.96	2.49	2.825 (2)	100
C8—H8*A*⋯O2^i^	0.96	2.40	3.217 (2)	142
C11—H11*C*⋯*Cg*1^ii^	0.96	3.00	3.748 (3)	136

Symmetry codes: (i) 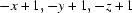; (ii) 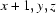.

**Fig. 1.**
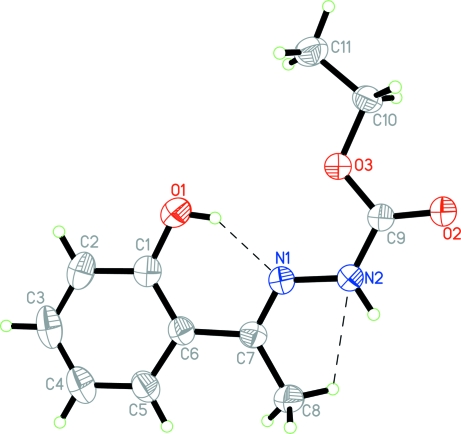
The molecular structure of the title molecule, with the atom-numbering scheme. Displacement ellipsoids are drawn at the 30% probability level. Hydrogen bonds are shown as dashed lines.

**Fig. 2.**
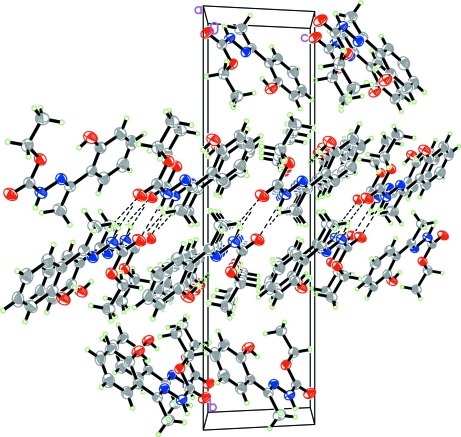
A packing diagram of the title compound. Hydrogen bonds are shown as dashed lines.

## supplementary crystallographic information

### Comment

Benzaldehydehydrazone derivatives have received considerable attention for a long time, due to their pharmacological activities (Parashar *et al.*, 1988) and their photochromic properties (Hadjoudis *et al.*,  1987). They are important intermidiates fot 1,3,4-oxadiazoles, which have been reported to be versatile compounds with many properties (Borg *et al.*,  1999). As a further investigation of this type of derivatives, we report herein the crystal structure of the title compound.

The title molecule (Fig. 1) adopts a trans configuration with respect to the C═N bond. The bond lengths and angles agree with those observed for (E)-ethyl N'-(4-bromobenzylidene)hydrazinecarboxylate (Gao, 2008). The ring A (C1-C6) is, of course, planar and it is oriented with respect to the mean plane of (C9/C10/C11/N1/N2/O2/O3) at a dihedral angle of 3.65 (12)°. The intramolecular C-H···N and O-H···N hydrogen bonds (Table 1) result in the formation of five- and six-membered rings: B (N1/N2/C7/C8/H8A) and C (N1/O1/H1/C1/C6/C7). Ring B adopts envelope conformation, with H8A atom displaced by -0.413 (3) Å from the plane of the other ring atoms. Ring C is nearly planar and it is oriented with respect to ring A at a dihedral angle of 2.38 (3)°.

In the crystal structure, intermolecular N-H···O and C-H···N hydrogen bonds (Table 1) link the molecules (Fig. 2), in which they may be effective in the stabilization of the structure. A C—H···π contact (Table 1) between the benzene ring and the methyl group further stabilize the structure.

### Experimental

2-Hydroxy-acetophenone (1.36 g, 0.01 mol) and ethyl hydrazinecarboxylate (1.04 g, 0.01 mol) were dissolved in stirred methanol (25 ml) and left for 4 h at room temperature. The resulting solid was filtered off and recrystallized from ethanol to give the title compound (yield; 85%, m.p. 487-489 K). Crystals suitable for X-ray analysis were obtained by slow evaporation of an ethanol solution.

### Refinement

H atoms were positioned geometrically, with O-H = 0.82 Å (for OH), N-H = 0.86 Å (for NH) and C-H = 0.93, 0.97 and 0.96 Å for aromatic, methylene and methyl H, respectively, and constrained to ride on their parent atoms with U_iso_(H) = xU_eq_(C,N,O), where x = 1.5 for OH H and methyl H and x = 1.2 for all other H atoms.

### Crystal data

**Table:**

C_11_H_14_N_2_O_3_	*F*_000_ = 472
*M**_r_* = 222.24	*D*_x_ = 1.290 Mg m^−^^3^
Monoclinic, *P*2_1_/*c*	Mo *K*α radiation λ = 0.71073 Å
Hall symbol: -P 2ybc	Cell parameters from 1018 reflections
*a* = 8.0841 (7) Å	θ = 1.8–25.0º
*b* = 23.052 (2) Å	µ = 0.10 mm^−^^1^
*c* = 6.6019 (6) Å	*T* = 273 (2) K
β = 111.584 (3)º	Block, colourless
*V* = 1144.03 (18) Å^3^	0.28 × 0.24 × 0.23 mm
*Z* = 4	

### Data collection

**Table:**

Bruker SMART CCD area-detector diffractometer	2018 independent reflections
Radiation source: fine-focus sealed tube	1360 reflections with *I* > 2σ(*I*)
Monochromator: graphite	*R*_int_ = 0.089
*T* = 273(2) K	θ_max_ = 25.0º
φ and ω scans	θ_min_ = 1.8º
Absorption correction: multi-scan(SADABS; Bruker, 2002)	*h* = −9→9
*T*_min_ = 0.973, *T*_max_ = 0.981	*k* = −27→25
12167 measured reflections	*l* = −7→7

### Refinement

**Table:**

Refinement on *F*^2^	Hydrogen site location: inferred from neighbouring sites
Least-squares matrix: full	H-atom parameters constrained
*R*[*F*^2^ > 2σ(*F*^2^)] = 0.040	*w* = 1/[σ^2^(*F*_o_^2^) + (0.0565*P*)^2^] where *P* = (*F*_o_^2^ + 2*F*_c_^2^)/3
*wR*(*F*^2^) = 0.117	(Δ/σ)_max_ = 0.004
*S* = 1.05	Δρ_max_ = 0.21 e Å^−^^3^
2018 reflections	Δρ_min_ = −0.12 e Å^−^^3^
149 parameters	Extinction correction: SHELXL97 (Sheldrick, 2008), Fc^*^=kFc[1+0.001xFc^2^λ^3^/sin(2θ)]^-1/4^
Primary atom site location: structure-invariant direct methods	Extinction coefficient: 0.003 (2)
Secondary atom site location: difference Fourier map	

### Special details

**Table:**

Geometry. All e.s.d.'s (except the e.s.d. in the dihedral angle between two l.s. planes) are estimated using the full covariance matrix. The cell e.s.d.'s are taken into account individually in the estimation of e.s.d.'s in distances, angles and torsion angles; correlations between e.s.d.'s in cell parameters are only used when they are defined by crystal symmetry. An approximate (isotropic) treatment of cell e.s.d.'s is used for estimating e.s.d.'s involving l.s. planes.
Refinement. Refinement of F^2^ against ALL reflections. The weighted R-factor wR and goodness of fit S are based on F^2^, conventional R-factors R are based on F, with F set to zero for negative F^2^. The threshold expression of F^2^ > 2sigma(F^2^) is used only for calculating R-factors(gt) etc. and is not relevant to the choice of reflections for refinement. R-factors based on F^2^ are statistically about twice as large as those based on F, and R- factors based on ALL data will be even larger.

### Fractional atomic coordinates and isotropic or equivalent isotropic displacement parameters (Å^2^)

**Table:**

	*x*	*y*	*z*	*U*_iso_*/*U*_eq_	
O1	0.48727 (18)	0.67615 (6)	−0.1086 (2)	0.0764 (4)	
H1	0.4714	0.6532	−0.0226	0.115*	
O2	0.33133 (16)	0.55003 (5)	0.4805 (2)	0.0700 (4)	
O3	0.30739 (14)	0.62476 (5)	0.25268 (17)	0.0577 (4)	
N1	0.54972 (18)	0.58512 (6)	0.1300 (2)	0.0547 (4)	
N2	0.50506 (17)	0.55461 (6)	0.2812 (2)	0.0575 (4)	
H2A	0.5583	0.5226	0.3333	0.069*	
C1	0.6180 (2)	0.65614 (8)	−0.1721 (3)	0.0596 (5)	
C2	0.6542 (3)	0.68841 (9)	−0.3299 (3)	0.0758 (6)	
H2	0.5901	0.7221	−0.3849	0.091*	
C3	0.7839 (3)	0.67078 (11)	−0.4046 (3)	0.0849 (7)	
H3	0.8064	0.6925	−0.5105	0.102*	
C4	0.8806 (3)	0.62129 (11)	−0.3238 (4)	0.0840 (6)	
H4	0.9690	0.6095	−0.3736	0.101*	
C5	0.8453 (3)	0.58970 (9)	−0.1696 (3)	0.0704 (5)	
H5	0.9112	0.5562	−0.1166	0.084*	
C6	0.7148 (2)	0.60525 (7)	−0.0876 (3)	0.0534 (4)	
C7	0.6823 (2)	0.56945 (7)	0.0794 (3)	0.0524 (4)	
C8	0.7998 (2)	0.51833 (8)	0.1808 (3)	0.0721 (6)	
H8A	0.7986	0.5114	0.3236	0.108*	
H8B	0.9192	0.5264	0.1915	0.108*	
H8C	0.7564	0.4846	0.0918	0.108*	
C9	0.3771 (2)	0.57519 (7)	0.3476 (3)	0.0521 (4)	
C10	0.1786 (2)	0.65209 (8)	0.3283 (3)	0.0606 (5)	
H10A	0.2313	0.6594	0.4837	0.073*	
H10B	0.0760	0.6271	0.3002	0.073*	
C11	0.1244 (3)	0.70765 (8)	0.2071 (3)	0.0771 (6)	
H11A	0.2266	0.7323	0.2384	0.116*	
H11B	0.0374	0.7267	0.2510	0.116*	
H11C	0.0744	0.6999	0.0535	0.116*	

### Atomic displacement parameters (Å^2^)

**Table:**

	*U*^11^	*U*^22^	*U*^33^	*U*^12^	*U*^13^	*U*^23^
O1	0.0824 (9)	0.0654 (9)	0.0859 (10)	0.0151 (7)	0.0364 (8)	0.0187 (7)
O2	0.0769 (8)	0.0633 (8)	0.0855 (9)	0.0117 (6)	0.0484 (7)	0.0207 (7)
O3	0.0619 (7)	0.0571 (8)	0.0613 (7)	0.0126 (6)	0.0310 (6)	0.0104 (6)
N1	0.0578 (8)	0.0550 (9)	0.0557 (8)	0.0027 (6)	0.0262 (7)	0.0052 (7)
N2	0.0613 (8)	0.0538 (9)	0.0651 (9)	0.0103 (7)	0.0323 (7)	0.0122 (7)
C1	0.0621 (10)	0.0590 (11)	0.0553 (10)	−0.0084 (9)	0.0188 (9)	−0.0026 (9)
C2	0.0857 (14)	0.0681 (13)	0.0640 (12)	−0.0163 (11)	0.0163 (11)	0.0106 (10)
C3	0.1013 (16)	0.1014 (18)	0.0584 (13)	−0.0409 (14)	0.0370 (12)	−0.0050 (12)
C4	0.0921 (15)	0.1005 (18)	0.0737 (14)	−0.0166 (14)	0.0471 (13)	−0.0061 (13)
C5	0.0756 (12)	0.0776 (14)	0.0682 (12)	−0.0021 (10)	0.0385 (10)	−0.0014 (10)
C6	0.0561 (10)	0.0547 (11)	0.0507 (10)	−0.0066 (8)	0.0211 (8)	−0.0052 (8)
C7	0.0522 (9)	0.0510 (10)	0.0548 (10)	0.0002 (8)	0.0207 (8)	−0.0033 (8)
C8	0.0722 (12)	0.0655 (13)	0.0891 (14)	0.0136 (10)	0.0420 (11)	0.0129 (10)
C9	0.0533 (9)	0.0509 (11)	0.0545 (10)	0.0019 (8)	0.0227 (8)	0.0032 (8)
C10	0.0572 (10)	0.0685 (12)	0.0602 (11)	0.0091 (8)	0.0266 (8)	−0.0005 (9)
C11	0.0907 (14)	0.0644 (13)	0.0770 (13)	0.0209 (11)	0.0319 (11)	0.0041 (10)

### Geometric parameters (Å, °)

**Table:**

O1—H1	0.8200	C7—N1	1.286 (2)
N2—N1	1.3735 (18)	C7—C8	1.507 (2)
N2—H2A	0.8600	C8—H8A	0.9600
C1—O1	1.353 (2)	C8—H8B	0.9600
C2—C3	1.374 (3)	C8—H8C	0.9600
C2—C1	1.396 (3)	C9—O2	1.2168 (18)
C2—H2	0.9300	C9—O3	1.3251 (19)
C3—C4	1.375 (3)	C9—N2	1.350 (2)
C3—H3	0.9300	C10—O3	1.4537 (19)
C4—H4	0.9300	C10—C11	1.488 (3)
C5—C4	1.365 (3)	C10—H10A	0.9700
C5—H5	0.9300	C10—H10B	0.9700
C6—C5	1.398 (2)	C11—H11A	0.9600
C6—C1	1.407 (2)	C11—H11B	0.9600
C6—C7	1.476 (2)	C11—H11C	0.9600
			
C1—O1—H1	109.5	N1—C7—C6	115.56 (14)
C9—O3—C10	116.40 (13)	N1—C7—C8	123.60 (15)
C7—N1—N2	120.80 (14)	C6—C7—C8	120.84 (14)
C9—N2—N1	119.60 (14)	C7—C8—H8A	109.5
C9—N2—H2A	120.2	C7—C8—H8B	109.5
N1—N2—H2A	120.2	H8A—C8—H8B	109.5
O1—C1—C2	116.86 (18)	C7—C8—H8C	109.5
O1—C1—C6	123.05 (16)	H8A—C8—H8C	109.5
C2—C1—C6	120.09 (18)	H8B—C8—H8C	109.5
C3—C2—C1	120.5 (2)	O2—C9—O3	124.27 (15)
C3—C2—H2	119.7	O2—C9—N2	122.61 (16)
C1—C2—H2	119.7	O3—C9—N2	113.11 (14)
C2—C3—C4	120.43 (19)	O3—C10—C11	107.12 (14)
C2—C3—H3	119.8	O3—C10—H10A	110.3
C4—C3—H3	119.8	C11—C10—H10A	110.3
C5—C4—C3	119.1 (2)	O3—C10—H10B	110.3
C5—C4—H4	120.4	C11—C10—H10B	110.3
C3—C4—H4	120.4	H10A—C10—H10B	108.5
C4—C5—C6	123.2 (2)	C10—C11—H11A	109.5
C4—C5—H5	118.4	C10—C11—H11B	109.5
C6—C5—H5	118.4	H11A—C11—H11B	109.5
C5—C6—C1	116.69 (16)	C10—C11—H11C	109.5
C5—C6—C7	120.86 (16)	H11A—C11—H11C	109.5
C1—C6—C7	122.45 (15)	H11B—C11—H11C	109.5
			
C9—N2—N1—C7	173.35 (15)	C5—C6—C7—N1	−174.51 (15)
C3—C2—C1—O1	−179.43 (16)	C1—C6—C7—N1	5.5 (2)
C3—C2—C1—C6	0.2 (3)	C5—C6—C7—C8	5.6 (2)
C1—C2—C3—C4	−0.4 (3)	C1—C6—C7—C8	−174.34 (16)
C2—C3—C4—C5	0.5 (3)	C6—C7—N1—N2	179.08 (13)
C6—C5—C4—C3	−0.3 (3)	C8—C7—N1—N2	−1.1 (2)
C5—C6—C1—O1	179.61 (16)	O2—C9—O3—C10	5.1 (2)
C7—C6—C1—O1	−0.4 (3)	N2—C9—O3—C10	−175.28 (14)
C5—C6—C1—C2	0.0 (2)	O2—C9—N2—N1	179.74 (14)
C7—C6—C1—C2	179.98 (15)	O3—C9—N2—N1	0.1 (2)
C1—C6—C5—C4	0.0 (3)	C11—C10—O3—C9	177.71 (14)
C7—C6—C5—C4	−179.95 (17)		

### Hydrogen-bond geometry (Å, °)

**Table:**

*D*—H···*A*	*D*—H	H···*A*	*D*···*A*	*D*—H···*A*
O1—H1···N1	0.82	1.85	2.5604 (18)	145
N2—H2A···O2^i^	0.86	2.07	2.9175 (18)	167
C8—H8A···N2	0.96	2.49	2.825 (2)	100
C8—H8A···O2^i^	0.96	2.40	3.217 (2)	142
C11—H11C···Cg1^ii^	0.96	3.00	3.748 (3)	136

Symmetry codes: (i) −*x*+1, −*y*+1, −*z*+1; (ii) *x*+1, *y*, *z*.
